# Rethinking Immunity against Pneumococcal Disease

**DOI:** 10.1371/journal.pmed.0020020

**Published:** 2005-01-25

Streptococcus pneumoniae is a common bacterium that is present in the nasopharynx of many children and some adults, where it causes no harm to its carrier but can be transmitted to others. If it moves beyond the nasopharynx, however, it can cause ear infections or invasive disease, such as pneumonia or meningitis. Invasive disease from this organism occurs especially in children, the elderly, and individuals with weakened immune systems.

The protective effect of antibodies against bacterial pneumonia has been appreciated since the 1930s, when it was shown that serum therapy—the transfer of serum from an immunized animal to a patient with acute disease caused by the same bacterial strain—could reduce mortality from pneumococcal pneumonia by half. Subsequent development of vaccines based on the bacterium's polysaccharide capsule, which could protect against infection, confirmed that an endogenous antibody response can provide protection against invasive disease.[Fig pmed-0020020-g001]

**Figure pmed-0020020-g001:**
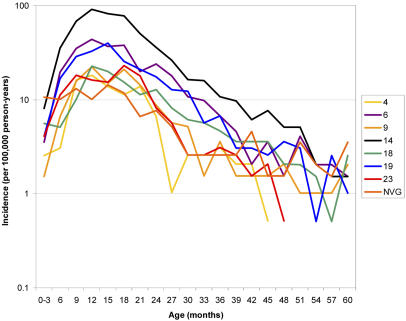
Parallel age-incidence curves for pneumococcal serotypes suggest a common mechanism of protection

One challenge for vaccine development has been the existence of many different serotypes (the same species of bacteria but with different composition of the polysaccharide capsule). As protection usually doesn't extend to different serotypes, vaccination with capsule components from different serotypes is necessary to ensure broad protection. Such vaccines have been shown to be efficient and safe. They are now recommended in many countries for infants and toddlers, and for people over 65—the two age groups in which invasive disease is most common—and for others who are at increased risk of pneumococcal disease (e.g., patients with heart, kidney, liver, or lung disease, or who have had a splenectomy).

Even without vaccination, however, most exposed individuals will never get invasive disease. Instead, they develop natural immunity against the different serotypes, though this immunity gradually declines with old age. Marc Lipsitch and colleagues wanted to understand the immunological basis of this natural immunity, and specifically whether it was due to anticapsular antibodies.

If protection from invasive disease is due to acquiring anticapsular antibodies against each of the pneumococcal serotypes, they argued, this would lead to two predictions about the age distribution of disease caused by the different serotypes in the non-vaccinated population. First, for serotypes that are more common and therefore encountered earlier in life, children should develop immunity more quickly, causing disease from these types to drop off earlier in life than disease from the less common types. Second, protection against invasive disease from a particular serotype should coincide with the acquisition of antibodies against that serotype, on both the individual and population level.

Neither prediction was borne out by the actual data the researchers analyzed, suggesting that there is more to natural immunity against pneumococcal disease than just anticapsular antibodies. The study doesn't demonstrate what the additional components are, but additional research might not just teach us about our immune system but also provide clues for further vaccine development. As the authors say, “A better understanding of the mechanisms that underlie natural immunity to pneumococcus could pave the way for the development of more effective, species-specific pneumococcal vaccines.”

