# Correction: Rethinking Immunity against Pneumococcal Disease

**DOI:** 10.1371/annotation/b9f2fdde-2923-4dfe-beff-b101d023f7b0

**Published:** 2012-03-05

**Authors:** Rethinking Immunity against Pneumococcal Disease

An image that was published alongside another PLoS Medicine article (doi/10.1371/journal.pmed.0020024) and included in the shared PDF download here has been removed from the published PDF because of that image's copyright status. 

